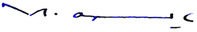# Building an international platform for the research, development, and dissemination of new knowledge in the field of laboratory animal science, technology, and experimental medicine

**DOI:** 10.1002/ame2.12570

**Published:** 2025-02-05

**Authors:** Muhammad Iqbal Choudhary

**Affiliations:** ^1^ Organization of lslamic Cooperation Standing Committee on Scientific and Technological Cooperation; ^2^ International Center for Chemical and Biological Sciences University of Karachi; ^3^ The University of Lahore; ^4^ The World Academy of Sciences

Dear Colleagues,

It is my distinct honor to serve as the Honorary Chief Editor of *AMEM*, an international open‐access journal, dedicated to serve as a platform for international exchange, and the translation of research outcomes in the field of life sciences. *AMEM* strives to establish itself as a high‐level international academic exchange platform that brings together scientific wisdom from around the world and offers a prestigious journal for presenting research findings, and disseminating state of the art knowledge in the vibrant fields of life sciences.

*AMEM*'s strong academic foundation and extensive international influence are evident from its inclusion in esteemed databases, such as ESCI, MEDLINE, Scopus, PMC, and DOAJ, with a Q2 ranking in JCI and JCR, and widespread indexing by academic search engines including Google Scholar. The articles published in *AMEM* cover various frontier areas of animal model preparation, laboratory animal science, and experimental medicine, featuring special columns on medical artificial intelligence, stem cells and regenerative medicine, neurodegenerative diseases, and cardiovascular and cerebrovascular disease research, making it one of the key platform for dissemination of new and novel research, and developments to global readers. I am sure the untiring and sincere efforts of the Editorial team and contributing authors will help the *AMEM* to leap forward to greater heights.

Here, I sincerely invite researchers in various fields of life sciences to contribute original articles to *AMEM*. Whether you specialize in the development and utilization of experimental animal resources or are focused on experimental medical research on cancers, neurodegenerative diseases, and more, *AMEM* is an ideal choice for showcasing your research findings and promoting academic excellence. We greatly appreciate that your scholarly contributions will play a pivotal role in advancing experimental animal, and experimental medicine fields.

I sincerely hope that researchers worldwide will join hands with the *AMEM* team in our shared mission of showcasing the latest research findings and academic excellence from the platform of *AMEM*. Our team will make sure that your research breakthroughs reach every corner of the world.

Finally, I wish all of you continued success, and look forward to witnessing the sustained growth in the stature and outreach of the *AMEM*.

Thank you all!

*AMEM* Honrary Editor‐in‐Chief